# Comparative Study on the Larvicidal Activity of Drimane Sesquiterpenes and Nordrimane Compounds against *Drosophila melanogaster til-til*

**DOI:** 10.3390/molecules18044192

**Published:** 2013-04-10

**Authors:** Ivan Montenegro, Luis Pino, Enrique Werner, Alejandro Madrid, Luis Espinoza, Luis Moreno, Joan Villena, Mauricio Cuellar

**Affiliations:** 1 Departamento de Química, Universidad Técnica Federico Santa María, Av. España N° 1680, Valparaíso 2340000, Chile; E-Mails: alejandro.madrid@usm.cl (A.M.); luis.espinozac@usm.cl (L.E.); 2 Departamento de Ciencias Básicas, Universidad del Biobío, Campus Fernando May, Avda. Andrés Bello s/n casilla 447, Chillán 3780000, Chile; E-Mails: luisandrespinocastro@hotmail.com (L.P.); lmoreno@ubiobio.cl (L.M.); 3 Escuela de Medicina, Centro de Investigaciones Biomédicas (CIB), Universidad de Valparaíso, Av. Hontaneda N° 2664, Valparaíso 234000, Chile; E-Mail: juan.villena@uv.cl; 4 Facultad de Farmacia, Universidad de Valparaíso, Av. Gran Bretaña N° 1093, Valparaíso 234000, Chile

**Keywords:** antifeedantactivity, *Drosophila melanogaster til-til*, nordrimanes, drimanes, sesquiterpenes

## Abstract

Natural compounds from *Drimys winteri* Forst and derivatives exhibited larvicidal effects against *Drosophila melanogaster til-til*. The most active compound was isodrimenin (**4**). The highest lethal concentration to the larvae of *D. melanogaster* was 4.5 ± 0.8 mg/L. At very low concentrations drimenol (**1**), confertifolin (**3**), and drimanol (**5**) displayed antifeedant and larvae growth regulatory activity. The antifeedant results of nordrimanic and drimanic compounds were better in first instar larvae. The EC_50_ value of polygodial (**2**) was 60.0 ± 4.2 mg/L; of diol **15** 45.0 ± 2.8 mg/L, and of diol **17** 36.9 ± 3.7 mg/L, while the new nordrimane compound **12** presented a value of 83.2 ± 3.5 mg/L.

## 1. Introduction

*Droshophila melanogaster* is a yellow-red dipterous (with two wings) 3–5 mm in size insect, usually with red eyes, also known as the vinegar fly or fruit fly. Its development cycle is very fast, and is favored by access to sugary liquids. The entire life cycle lasts between 9 and 20 days, and in summer it is approximately 7 to 8 days. The females come to lay 700 to 800 eggs during their lifetime. The egg-laying takes place on wounded fruits, and liquids like juices, vinegar, wine, beer and soft drinks. The larvae are tiny worms, whitish in appearance, that can be found inside the damaged fruit. The main damage caused by this insect is due to its ability to transmit diseases such as sour rot, recognizable by the strong vinegar smell emit by contaminated items. The til-til strain proved to be a new species of *Drosophila* belonging to the funebris group [1,2,3].

Since the discovery of drimenol (1), the first drimane sesquiterpenoid, in *Drimys winteri* f. bark by Appel on 1948 [4], hemisynthetic derivatives of this natural compound have been prepared. At a biological level, drimenol has a similar function to the heteroauxin indoleacetic acid, a growth promoting hormone occurring in some plants [5]. However, its most important feature from the organic synthesis viewpoint is its utility as a starting compound for the preparation of biologically active drimanes and nordrimanes. Among the biological activities of drimanic compounds are their antimicrobial, antifungal, growth inhibitors, molluscicide and gastroprotective properties [6,7,8,9]. For example, ugandensidial is a strong antifungal and warbunganal, another drimane dialdehyde naturally found in *Warburgia ugandensis*, is used in Africa for its various properties, which include antiallergy, antibacterial, antifungal and antifeedant activities, and can be synthesized using drimenol (1) as starting material [5,10,11].

## 2. Results and Discussion

### 2.1. Chemistry

**Scheme 1:**
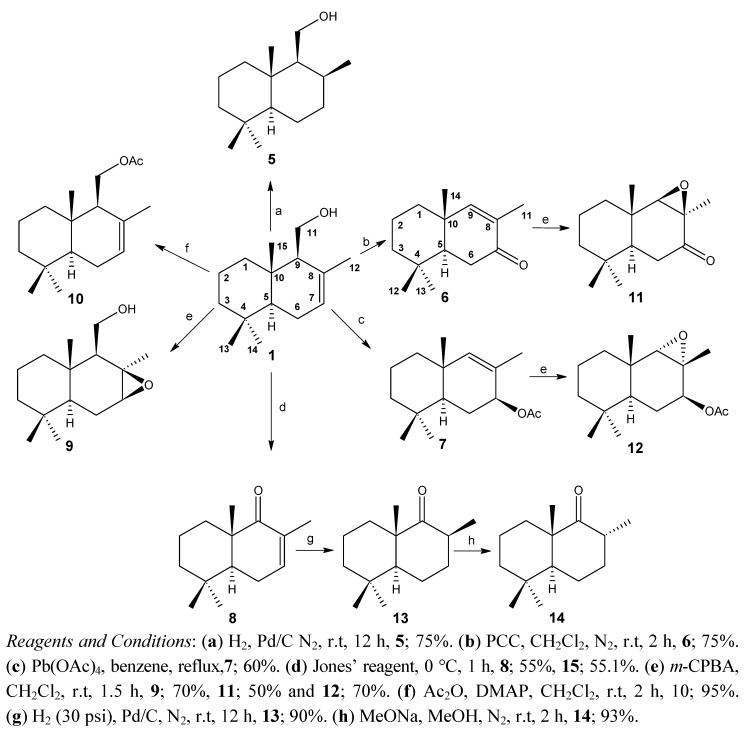
Synthesis of drimane and nordrimane derivatives of drimenol (1).

The primary defence compound in *P. colorata* leaves is known—it is the sesquiterpene dialdehyde polygodial (2) which imparts a pungent taste and has potent insect antifeedant properties [12]. *In vitro* studies have shown polygodial was an effective antifeedant at concentrations of 3 mg/g [13]. In this paper, we report the synthesis of two new nordrimanes and five known nordrimanes from drimenol (1) which were then compared with eleven sesquiterpene compounds for activity against *Drosophila melanogaster til-til*. The compounds 5–10 and 13–14 were synthesized from drimenol (1) using standard methods. The new epoxides 11 and 12 were synthesized from 6 and 7, respectively. This was performed through the *m*-CPBA epoxidation protocol producing a 50% yield for 11 and a 70% yield for 12, respectively (Scheme 1).

The catalytic hydrogenation of the double endocyclic bond of compound 8 was carried out using H_2_ and Pd/C as previously described for other alkenes [14]. This takes place side via the less sterically hindered α-face, to give 13 in 90% yield (Scheme 1). The methyl group is positioned on the β face. By treating 13 with sodium methoxide in methanol, an epimerization of the CH_3_ group occurs, as evidenced by the fact that the axial position changed to equatorial giving ketone 14. By comparing the ^13^C-NMR spectra of 13 and 14, it can be seen that carbonyl signal shift changed from 220.5 to 216.7 ppm, respectively. All the compounds were characterized by their IR, NMR and MS spectral data. 

*Reagents and Conditions*: (a) H_2_, Pd/C N_2_, r.t, 12 h, 5; 75%. (b) PCC, CH_2_Cl_2_, N_2_, r.t, 2 h, 6; 75%. (c) Pb(OAc)_4_, benzene, reflux,7; 60%. (d) Jones’ reagent, 0 °C, 1 h, 8; 55%, 15; 55.1%. (e) *m*-CPBA, CH_2_Cl_2_, r.t, 1.5 h, 9; 70%, 11; 50% and 12; 70%. (f) Ac_2_O, DMAP, CH_2_Cl_2_, r.t, 2 h, 10; 95%. (g) H_2_ (30 psi), Pd/C, N_2_, r.t, 12 h, 13; 90%. (h) MeONa, MeOH, N_2_, r.t, 2 h, 14; 93%.

The identities of the other compounds 5–10 and 13,14 were established by comparison with the previously reported spectral data for the same compounds [15,16,17,18,19,20,21]. Epoxidation of 6 with *m*-chloroperbenzoic acid produced a single epoxide 11 in 70% yield. This reaction was performed under standard epoxidation conditions. The structure was determined by the ^1^H-NMR spectrum, where a shift in the H-9 signal from δ = 6.22 to δ = 3.35 ppm was observed [22].

**Figure 1:**
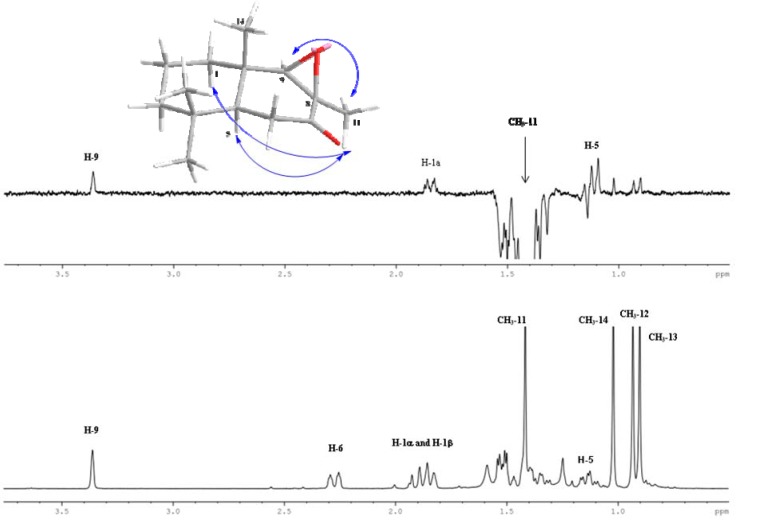
Bottom: ^1^H-NMR spectrum of compound 11. Top: 1D ^1^H sel. gs NOESY experiment (*selnogp* pulse program from Bruker Library) showing the main long-range interactions between CH_3_-11 and H-5, H-1α and H-9.

The assignment of the configuration in C-8 and C-9 for compound 11 was proposed on the basis of NOE-spectra observations. From a ^1^H-1D sel. gs-NOESY experiment, when CH_3_-11 was selectively irradiated only long-range interactions with H-9, H-1α and H-5 were observed. In addition, no long-range interactions between H-9 with the CH_3_-14 were observed. Thereby this NMR experiment confirms the β spatial orientation of the epoxide ring and the stereochemistry of C-8 and C-9, according to Figure 1.

**Figure 2:**
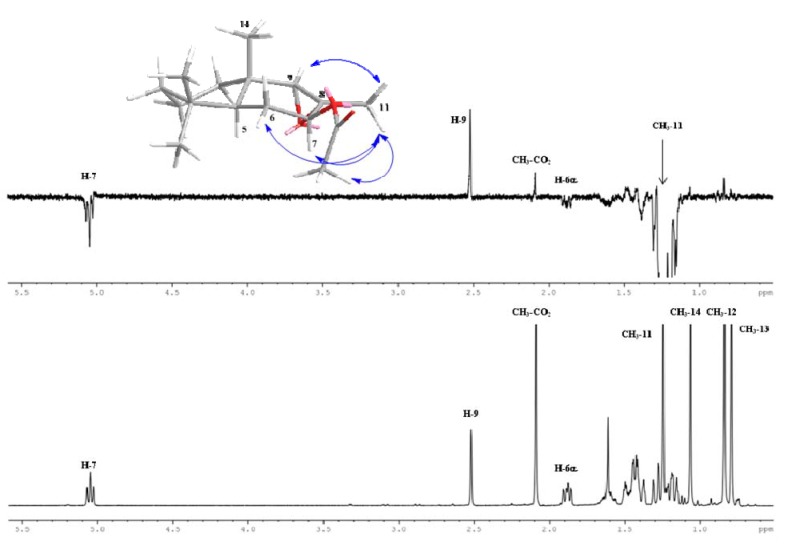
Bottom: ^1^H-NMR spectrum of compound 12. Top: 1D ^1^H sel. gs NOESY experiment (*selnogp* pulse program from Bruker Library) showing the main long-range interactions between CH_3_-11 and H-9, and CH_3_CO-9. Three spins NOE effect was also observed between CH_3_-11 with H-7 and H-6α (in negative phase).

On the other hand, from the 1H-1D sel. gs-NOESY experiment for compound 12, when CH_3_-11 was selectively irradiated long-range interactions with H-9 and CH_3_CO were observed. Additionally due to the conformation of ring B in the molecule, a long-range interaction, probably a three spin system with H-6α and H-7, was also observed (see Figure 2). In compound 12 H-9 has a NOE interaction with H-11 of the methyl group but it did not presented a NOE interaction with the H-7 which is placed in the α direction, which confirms the β direction of the methyl group on C-11 and the α stereochemistry of the epoxide group. This stereochemistry is explained by considering el steric hindrance caused by the acetate group, redirecting below the epoxidation reactions. Additionally shifts of C-8 and C-9 in compound 12 were compared with a compound of similar structure [23].

**Scheme 2:**
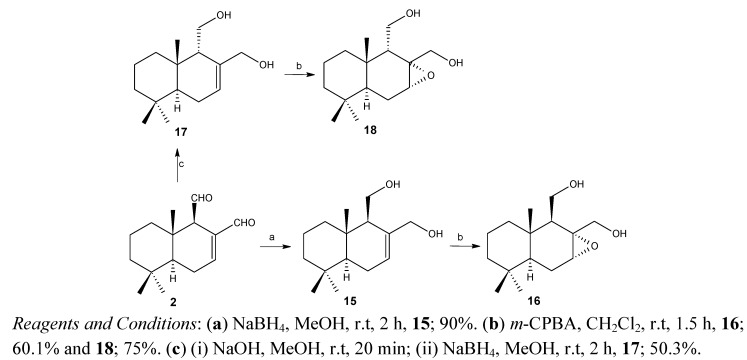
Synthesis of drimane derivatives of polygodial (2).

The compounds 15–18 were synthesized via the common starting material polygodial (2) as detailed below (Scheme 2). Their structures were established by comparison with the previously reported spectral data for the same compounds [24,25].

*Reagents and Conditions*: (a) NaBH_4_, MeOH, r.t, 2 h, 15; 90%. (b) *m*-CPBA, CH_2_Cl_2_, r.t, 1.5 h, 16; 60.1% and 18; 75%. (c) (i) NaOH, MeOH, r.t, 20 min; (ii) NaBH_4_, MeOH, r.t, 2 h, 17; 50.3%.

### 2.2. Biological Results

In our program designed to discover more active compounds from *Drimys winteri* and derivatives it was found that the compounds showed larvicidal activity. Based on this information and knowing that this tree has metabolites with a high resistance to insect and pathogen attack we have carried out a larvae growth regulatory study. The compounds were first used in a bioassay trial, and subsequently, in order to obtain more satisfactory data, they were used in as low concentrations as possible to establish the larvicidal activity, *ergo* there was no effect due to the toxicity of the samples to many targets that may not be specific to the insect but general or broad spectrum for plants, insects and humans.

### 2.3. Antifeedant Activity No-Choice Test

Some of the compounds showed a feeding dissuasive activity against *D. melanogaster* larvae in choice assays. The larvae treated with compounds 1, 3, 4, 15–17 showed moderate levels of feeding inhibition compared to control, showing a decrease in the weight at low concentrations of drimane compound (see Table 1). It is considered that significant feeding inhibition occurs when the antifeedant index (AI) is superior to 75% and moderate when it is between 50 and 75%. All samples were evaluated at 100, 50, 25, and 5 mg/L. The most active compound against *D. melanogaster* was 17, which showed high antifeedant effects (see Table 1), even at concentrations of 5 ppm (77.51%), while the effect was reduced to moderate on the next concentration at 25 ppm (59.41%). Isodrimenin (4) showed moderate effects, as well, with values between 5.0 and 25.0 ppm. Polygodial (2) has no significant antifeedant activity against the tested strain of *Drosophila melanogaster*, and although previous studies established it as a good insect antifeedant, we did not get a strong effect on larvae*.* Polygodial (2) had significative antifeedant activity as reported by Messchendorp *et al*. [26], who stated that drimanes are inactive at concentrations of less than 5 mM. We assayed a range from 21.4 µM to 2.14 mM. Only drimane compounds possessing a lactone group inhibited feeding in the no-choice tests. An exception is warburganal. In a previous work [27] Messchendorp *et al*. reported that the drimanes with a lactone group on the B-ring appear to be the most potent antifeedants at 5 mM (they tested a range from 1 mM to 5 mM). We observed antifeedant activity with isodrimenine (4) at 5 ppm which is equivalent to 21.2 µM, as shown in Table 1. It has been established thatfor polygodial(2) and other drimanic aldehydes, thevariation in the antifeedant activity of these compounds is consistent with the proposed mode of action for antifeedant drimanes, that is, adduct formation with amino groups on the insects’ molecular targets [24,28,29]. On the other hand, this is the first report of activity of those compounds on *Drosophila melanogaster*, and it is not possible to compare different kinds or species of insects, *i.e.*, *Spodoptera frugiperda* [24] with the fruit fly since phylogenetically they are very distant and furthermore *Drosophila* possess strong CytP450 activity [30]. For example, overexpression of a single P450 gene, Cyp6g1, is sufficient to confer DDT resistance in Drosophila [31]. This emphasis on adaptive responses to xenobiotics arises from the importance of insecticide resistance, which remains the main impediment for effective crop protection and the control of insect-borne diseases [32]. The Nrf2 ortholog cap ‘n’ collar isoform-C (CncC) has been identified as a central regulator of xenobiotic responses in *Drosophila*. Nrf2 plays an important role in regulating cellular defenses against oxidative and electrophilic stress (Nguyen *et al*. [33], Sykiotis and Bohmann [34]). The remaining compounds have no significant antifeedant activity. 

**Table 1:** Results obtained in antifeedant tests, using different concentrations of compounds on *D. melanogaster larvae*.

Concentration (ppm)	AI ** (Mean ± SD)	1	3	4	15	16	17
Control solvent	0.0						
5		51.82 ± 6.7	52.25 ± 5.4	53.00 ± 0.9	44.79 ± 3.3	53.98 ± 3.6	77.51 ± 2.7
25		45.18 ± 3.4	43.77 ± 6.2	31.55 ± 4.2	37.68 ± 3.6	29.46 ± 3.9	59.41 ± 1.7
50		34.27 ± 3.0	32.63 ± 2.3		32.63 ± 5.3	24.98 ± 6.3	41.63 ± 4.2
100		18.17 ± 1.2	16.40 ± 1.9		16.50 ±2.8	20.80 ± 2.2	31.32 ± 2.8
* Safrole 5	33.21 ± 0.8						
* Azadirachtin 5	53.26 ± 1.6						

The values followed by the same letter are not significantly different. The significance level *p* < 0.05. ** AI (%) = antifeedant index; AI = [(C−T)/(C+T)] × 100. * are (+)-ve controls.

### 2.4. Larvicidal Activity against *D. melanogaster*

The mortality at 168 h and EC_50_ values during the first 6 days for compounds tested in no-choice experiments against first instar larvae of *D. melanogaster*, are shown in Table 2, respectively. The compounds 2, 4, 7, 12, 15–17 present larvicidal activity at 48 h (data not shown). Surviving larvae pupate later and undergo eclosion, giving rise to normal insects. The larvicidal assay was evaluated over a 6 day period because the biological cycle from larva to pupa in *D*. *melanogaster til-til* requires 7 days. Polygodial (2), had an EC_50_ of 60.0 ± 4.2 mg/L, exhibiting 50% mortality at 168 h. Isodrimenin (4) showed a 100% mortality and the lowest EC_50_ (4.5 ± 0.8 mg/L), but its isomeric form confertifolin (3) had a lower mortality, only 4.1%. Compound 7 showed 33.3% mortality at 168 h. The new nordrimanic compound 12 presented a moderate activity (12.5% mortality and EC_50_ of 83.2 ± 3.5 mg/L). The diol 15 and 17 prepared from 2 caused a similar mortality effect, but the EC_50_ values were not similar, because 17 presented a high 70.9 ± 3.7 mg/L values in comparison with compound 15 (45.0 ± 2.8 mg/L). This is the first time that the larvicidal activity of drimanic derivatives was measured. Previous studies have reported that molecules having sesquiterpenic skeletons with an α,β-unsaturated systems showed strong insecticidal activity on larvae of *Drosophila melanogaster* [35].

**Table 2:** Mortality percentage of *D. melanogaster larvae*, after 168 h of the application of the compounds at different concentrations in larvae’s diet *D. melanogaster til-til*.

Compounds	% Mortality	EC_50_ (mg/L)
1	15.0 ± 0.3	>100
2	50.0 ± 0.4	60.0 ± 4.2
3	4.10 ± 0.9	>100
4	100 ± 0.2	4.5 ± 0.8
5	0.0	>100
6	4.2 ± 1.2	>100
7	33.3 ± 1.1	57.8 ± 2.3
8	0.0	>100
9	0.0	>100
10	0.0	>100
11	0.0	>100
12	12.5 ± 1.2	83.2 ± 3.5
13	0.0	>100
14	0.0	>100
15	46.2 ± 1.3	45.0 ± 2.8
16	35.3 ± 0.2	70.0 ± 2.6
17	35.0 ± 1.1	70.9 ± 3.7
18Safrole *Azadirachtin *	0.020.0 ± 1.636.7 ± 0.5	>10018.0 ± 1.515.0 ± 1.1

* (+)-ve controls.

### 2.5. Growth Inhibition and Relative Growth Index for *D. melanogaster til-til*

The growth index (GI or number of surviving larvae/total larvae used) and relative growth index (RGI or GItreated/GIcontrol) (Table 3) shown that the strongest effects correspond to compound 4 between 5.0 and 25.0 ppm (RGI 0.26 at 5 ppm and RGI 0.12 at 25 ppm). Compound 4 caused a strong decrease in the number of larvae that reach pupation (45% at 5.0 ppm). The percentage of larvae that reached pupation also decreased drastically with 15 (see Table 4) but the active concentration is different with compared to 4 (100, 250 and 500 ppm). Other compounds assayed exhibited a moderate activity, but the percentage of emergence of adults from the pupae was also drastically affected by increasing the concentration (see Table 4)

**Table 3:** GI and RGI of *D. melanogaster til-til* as a function of increased concentrations of most active compounds from *D. winteri* and derivates.

Compounds	Concentration(ppm)	GI^x^ RGI^z^	
Control solventSafrole *1Azadirachtin *2	0.05.05.0	0.95 ± 0.03 x 0.40 ± 0.02 x 0.19 ± 0.03 x	1.00.470.20
2	25.050.0100.0250.0500.0	0.92 ± 0.04 y0.86 ± 0.05 y0.79 ± 0.08 y0.61 ± 0.19 y0.32 ± 0.09 y	0.970.910.830.640.34
4	5.025.050.0 *	0.25 ± 0.03 y0.11 ± 0.09 y-	0.260.12-
7	25.050.0100.0250.0500.0	0.95 ± 0.12 y0.90 ± 0.13 y0.73 ± 0.10 y0.62 ± 0.09 y0.29 ± 0.06 y	1.00.950.770.650.31
15	25.050.0100.0250.0500.0	0.63 ± 0.07 y0.50 ± 0.09 y0.35 ± 0.12 y0.15 ± 0.04 y0.09 ± 0.05 y	0.660.530.370.160.09
17	25.050.0100.0	0.56 ± 0.12 y0.48 ± 0.10 y0.31 ± 0.06 y-	0.590.500.33-

x Mean of three replicates. y Means followed by the same letter within a column after ± standard error values are not significantly different in a Student–Newman–Keuls (SNK) test at *p* < 0.05 (treatments are compared by concentration to control), 95% confidence limits. z RGItreatment = GItreated/GIcontrol (for details please see the Experimental Section). * this concentration is more toxic to larvae.* are (+)-ve controls.

**Table 4:** Activities of compounds isolated from *Drimys winteri* F., compared with derivative compounds in this paper.

Treatment	Doses(mg/L)	Mean weight gained (mg) ^c^	% ^d^	Pupation(SP) ^e^	Emergence(%) ^f^	Mortality(%)
Control solvent	0.0	0.813 ± 0.15 a	100	100	100	0
Safrole *Azadirachtin *1	55550100	0.270 ± 0.16 a0.433 ± 0.13 a0.258 ± 0.14 a0.398 ± 0.17 a0.563 ± 0.18 a	33.253.332.049.069.0	10047.570.078.380.6	10063.378.076.377.4	20.036.715.0
2	550	0.59 ± 0.16 a0.647 ± 0.14 a	72.679.6	72.780.0	98.097.0	50.0
	100	0.698 ±0.12 a	85.9	86.0	98.0	
	5	0.255 ± 0.13 a	31.4	80.0	70.0	
3	50	0.413 ± 0.14 a	50.8	85.0	85.0	4.1
	100	0.587 ± 0.11 a	72.2	80.0	80.0	
	5	0.250 ± 0.10 a	30.7	45.0	46.7	
4	50	0	0	0	0	100
	100	0	0	0	0	
	5	0.810 ± 0.10 a	99.6	80.0	78.3	
6	50	0.797 ± 0.13 a	98.0	80.0	93.5	4.2
	100	0.800 ± 0.12 a	98.4	85.0	86.7	
	5	0.801 ± 0.08 a	98.5	91.6	95.0	
7	50	0.763 ± 0.09 a	93.8	95.0	95.0	33.3
	100	0.750 ± 0.09 a	92.3	73.3	97.8	
	5	0.803 ± 0.09 a	98.8	100	98.3	
12	50	0.793 ± 0.10 b	97.5	100	91.7	12.5
	100	0.769 ± 0.12 a	94.6	100	91.7	
	5	0.310± 0.08 a	38.3	80.0	80.0	
15	50	0.413± 0.08 a	50.8	70.0	80.0	46.2
	100	0.587 ± 0.06 a	72.2	95	80.0	
	5	0.243 ± 0.12 a	29.8	60.0	60.0	
16	50	0.443 ± 0.08 b	54.5	91.6	90.0	35.3
	100	0.533 ± 0.08 a	65.5	58.3	50.5	
	5	0.103 ± 0.11 a	12.6	93.3	93.5	
17	50	0.207 ± 0.12 a	25.4	85.0	85.0	35.0
	100	0.480 ± 0.15 a	59.0	65.0	91.5	

* (+)-ve controls. (a) The values for growth bioassay were from weight, values taken at 8 ± 1 days before pupation, the criteria followed was to account larvae that formed pupae, the larvae that not formed pupae were counted as died larvae. (b) Values taken after pupation. (c) Means followed by the same letter within a column after ± standard error values are not significantly different in a Student–Newman–Keuls (SNK) test at *p* < 0.05 (treatments are compared by concentration to control), 95% confidence limits. Mean of three replicates. (d) Percentage with respect to control. (e) SP: Survival Pupation = number of surviving pupa e × 100/total larvae for pupation. (f)% = number of adults emerged × 100/total number of pupae. (g) As control was used a normal diet with solvent only.

In some compounds (1, 3 and 15) we can observe a decrease in the effect upon increasing the dose, which has been consistent in all replicates, requiring further studies to elucidate the possible mechanisms, both chemical and physiological. Xenobiotic metabolizing systems for a given xenobiotic chemical are very complex, and a number of nuclear receptors, transcription factors, genes, and enzymes may be involved in its metabolism and detoxification [36,37]. A single xenobiotic molecule may be modified by multiple enzymes on multiple functional groups, producing multiple metabolites [36]. We postulate that the activation of xenosensors that induce metabolizing enzyme expression at low stressor doses, may overreact to some extent, resulting in a net response that transforms the added compound into a highly toxic substance and at higher doses, the compensatory mechanism is overwhelmed, leading to a reversal of the response [38].

## 3. Experimental

### 3.1. General

Unless otherwise stated, all purchased chemical reagents (Merck or Aldrich) were of the highest commercially available purity and were used without previous purification. Safrole and azadirachtin was obtained commercially from Sigma-Aldrich. IR spectra were recorded as thin films on a Nicolet 6700 FT-IR spectrometer and frequencies are reported in cm^−1^. ESI-MS/MS data was collected using a high resolution hybrid quadrupole (Q) and orthogonal time-of-flight (TOF) mass spectrometer (Micromass Q-Tof, Manchester, UK) with constant nebulizer temperature of 80 °C. The ESI source and the mass spectrometer were operated in a negative ion mode, and the cone and extractor potentials ware of 10 eV, with a scan range of *m/z* 100–500. The band infused into the ESI source at flow rates of 5 μL min^−1^ was dissolved in acetonitrile ion-induced dissociation (CID) with argon in the collision chamber. The values expressed are average mass and correspond to the [M-H] (University of Talca). ^1^H, ^13^C, ^13^C DEPT-135, sel. *gs* 1D ^1^H NOESY, *gs* 2D HSQC and *gs* 2D HMBC spectra were recorded in CDCl_3_ solutions and are referenced to the residual peaks of CHCl_3_ at *δ* = 7.26 ppm and *δ* = 77.0 ppm for ^1^H and ^13^C, respectively, on a Bruker Avance 400 Digital NMR spectrometer, operating at 400.1 MHz for 1H and 100.6 MHz for ^13^C. Silica gel (Merck 200–300 mesh) was used for column chromatography (C.C.) and HF-254 silica gel plates were used for TLC. TLC spots were detected by heating after spraying with 25% H_2_SO_4_ in H_2_O.

### 3.2. Plant Material

The stem bark of *D. winteri* adult trees was collected at the Malleco Province (Chile), in February 2010. A voucher specimen (N° Dw-10112) was deposited at the Herbarium of the Natural Products Laboratory, “Dr. Herbert Appel A.”, Department of Chemistry, Universidad Técnica Federico Santa María, Valparaíso, Chile. Fresh bark was carefully washed with abundant distilled water to remove any residue. Afterwards, it was dried in an oven at 35 °C to a constant weight. Once dried, it was stored in hermetically sealed plastic containers at 4 °C.

### 3.3. Obtainment of Natural Compounds 1–4

**Figure 3:**
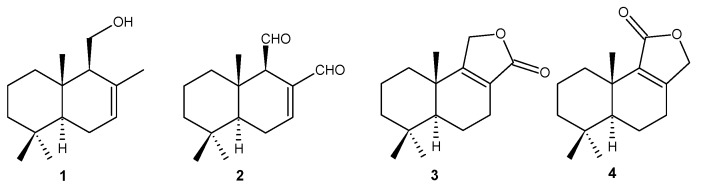
Compounds characterized from the *D. winteri* dichloromethane extract.

The natural drimanes drimenol (1), polygodial (2), confertifolin (3) and isodrimenin (4) (Figure 3) were isolated from the dichloromethane extract of *D. winteri* (Winteraceae) bark. The extraction methodology and isolation of pure compounds was performed according to reported procedures [24,39]. Compounds 1–4 were identified by melting point, optical rotation and spectroscopic data including ^1^H- and ^13^C-NMR and comparisons with data reported in the literature [39,40,41].

### 3.4. Preparation of Drimenol Derivatives 5–14

Compounds 5–10 and 14 were synthesized by treating drimenol (1) using different protocols reported in the literature [15,42,43]. The synthesis of compounds 11–13 is described below: 

*Synthesis of (5S,8R,9R,10S)-4,4,8α,10β-tetramethyloctahydronaphtho[8,9-β]oxiren-7)-one* (11) from 6. The enone 6 (1.0 g, 4.8 mmol) was treated in CH2Cl2 (30 mL) with m-CPBA (0.94 g, 5.4 mmol). Then the mixture was stirred for 1 h at room temperature. After this, the solution was taken up in CH2Cl2 (20 mL) and washed with saturated NaHCO_3_ solution. Later, the organic layer was dried over MgSO_4_, filtered and evaporated. Then it was absorbed on silica and subjected to C.C. eluting with mixtures of petroleum ether/EtOAc of increasing polarity (20.0:0.0→18.0:2.0) to give an colorless oil identified as epoxide 11 (0.26 g, 24.8%); [α]_D_^27^ = +0.5° (c = 14.58, CHCl_3_); IR (cm^−1^): 1699; 1452; 1251. ^1^H-NMR: 3.35 (s, 1H, H-9); 2.27 (m, 2H, H-6); 1.84 (m, 2H, H-1); 1.52 (m, 3H, H-2 and H-5); 1.41 (s, 3H, H-11); 1.14 (m, 2H, H-3); 1.01 (s, 3H, H-14); 0.92 (s, 3H, H-12); 0.89 (s, 3H, H-13). ^13^C-NMR: 208.2 (C-7); 59.5 (C-9); 56.2 (C-8); 46.1 (C-10); 41.4 (C-3); 39.0 (C-5); 33.2 (C-4); 32.7 (C-2); 32.6 (C-13); 22.5 (C-12); 22.0 (C-1); 17.8 (C-6); 16.9 (C-14); 16.8 (C-11). HREIMS: M+H ion *m*/*z* 223.1619 (calcd. For C_14_H_22_O_2_: 222.3286). 

*Synthesis of (5S,7S,8R,9S,10S)-4,4,8β,10β-tetramethyldecahydronaphtho[8,9-α]oxiren-7-yl* acetate (12) *from* 7. Compound 7 (1.0 g, 3.99 mmol) was treated in CH2Cl2 (30 mL) with m-CPBA (0.94 g, 5.4 mmol). Then the mixture was stirred for 1 hour at room temperature. After this, the solution was taken up in CH2Cl2 (20 mL) and then washed with saturated NaHCO3 solution. Later, the organic layer was dried over MgSO4, filtered, and evaporated. Then it was absorbed on silica, and subjected to C.C. eluting with mixtures of petroleum ether/EtOAc of increasing polarity (20.0:0.0→18.0:2.0) to give an white solid identified as epoxide 12 (0.74 g, 68.0%.); p.f.: 68.5–70.0 °C. [α]_D_^27^ = +0.36° (c = 14.58, CHCl_3_); IR (cm^−1^): 1779; 1452; 1254. ^1^H-NMR: 5.05 (t,1 H, *J* = 8.8 Hz, H-7); 2.52 (s, 1H, H-9); 2.09 (s, 3H, CH_3_); 1.88 (m, 1H, H-6α); 1.60 (m, 2H, H-4 and H-6 β); 1.47 (m, 4H, H-1 and H-2); 1.24 (s, 3H, H-11); 1.18 (m, 2H, H-3); 1.06 (s, 3H, H-14); 0.84 (s, 3H, H-12); 0.79 (s, 3H, H-13). ^13^C-NMR: 170.4.2 (CH_3_CO); 71.5 (C-7); 70.7 (C-9); 58.9 (C-8); 4154 (C-5); 39.9 (C-3); 36.3 (C-1); 34.5 (C-5); 33.0 (C-10); 32.5 (C-13); 25.0 (C-12); (CH_3_CO); 21.1 (C-2); 19.5 (C-14); 18.2 (C-11). HREIMS: M+H ion *m*/*z* 267.1882 (calcd. For C_16_H_26_O_3_: 266.3819).

### 3.5. Preparation of Polygodial Derivatives 15–18

Compounds 15–18 were synthesized by treating polygodial (2) using different protocols reported in the literature [25].

### 3.6. Fly Strains

Wild *Drosophila melanogaster til-til*, captured in the town of Til-Til metropolitan region, were grown in a Burdick culture medium, with propanoic acid as antifungal. Cultures were observed daily with a ZEISS Stemi DV4 binocular loupe to analyze the developmental stages of eggs and first instar larvae. Later, adult flies were used to deposit their eggs in medium and grown in a MEMMERT Model UM-500 incubation chamber at 25 °C for 4 days. 

### 3.7. No-Choice Test (Insecticidal Bioassay) against D. melanogaster

The bioassay for insecticidal activity against larvae of *D. melanogaster* was carried out as follows: six concentrations (10.0, 25.0, 50.0, 100.0, 250.0 and 500.0 ppm of sample) were used for determining EC_50_ values. For the bioassay test compounds were dissolved in 50 µL of acetone and mixed in 1 mL of artificial diet containing (for 1 kg of diet): sterile water (800 mL), agar (10.0 g), soy meal (50.0 g), corn meal (96.0 g), yeast extract (40.0 g), wheat germ (4.0 g), sorbic acid (2.0 g), choline chloride (2.0 g), ascorbic acid (4.0 g), *p*-hydroxybenzoic acid methyl ester (2.5 g), Wesson salt mixture (7.0 mL), Vanderzant vitamin mixture for insects (15.0 mL), formaldehyde (2.5 mL), streptomycin (0.1 unit), aureomycin (5.0 g), and milled corn grain ears (20.0 g), which was prepared by the procedure described earlier (Mihm [44]). Safrole and azadirachtin was used as a positive control at 5 ppm. A control diet was treated with 50 µL of acetone only. About 100 adults of D. melanogaster were introduced into a culture bottle with artificial diet, were subsequently transferred into a Petri dish, and allowed to oviposit at 25 °C and relative humidity > 60% for 3 h. The diet was taken out of the bottle, and 20 new eggs were collected and transplanted onto each diet (1 mL) in glass tubes and reared at 25 °C and relative humidity >90% for 8 days. One day after transplantation, the larvae were hatched and fed each test compound with the artificial diet [45]. In each instance, the developmental stage was observed, and the numbers of pupae were recorded and compared with those of a control. Ten new eggs were used in each of three replicates. The EC_50_, median effective concentrations, was determined by log-PROBIT analysis.

### 3.8. Relative Growth Index and Growth Index

The relative growth index (RGI) and growth index (GI) were calculated according to the literature [46], where: RGI = GI_treated_/GI_control_, GI_treated_ = number of surviving larvae after treatment, GI_control_ = total larvae used.

#### 3.8.1. Antifeedant Test against D. melanogaster

To determine the amount of larvae feeding, tests were carried out as follows a four concentrations (100, 50, 25, and 5 ppm of sample) and two controls were taken, one with acetone and the other without any treatment as a check. Twenty larvae previously weighed about at the transition between the second and third terms were placed on the diet (the same diet of the larvicidal assay) in each container for each assay. Based on the amount of food consumed, the absolute deterrence coefficient was calculated using the formula according to Kielczewski *et al*. [47]: 

coefficient AI* = (C − T): (C + T) × 100 (1)

where T is weight of food eaten by larvae in the experimental variant with compound and C is the weight of food consumed in the control variant. The changes in the body weight of tested larvae and the amount of eaten food per mg of body weight increase were also noted. After 48 h, the larvae received fresh diet every 24 h [48]. The first control group was treated with acetone and the second control group without any treatment for the first 48 h and then with fresh diet every 24 h, and finally dead larvae were removed [49].

#### 3.8.2. Statistical Analyses

Data shown in tables are average results obtained by means of three or 10 replicates and are presented as average ± standard errors of the mean (SEM). Data were subjected to analysis of variance (ANOVA) with significant differences between means identified by GLM procedures. Results are given in the text as probability values, with *p* < 0.05 adopted as the criterion of significance. Differences between treatment means were established with a Student–Newman–Keuls (SNK) test. The EC_50_ values for each activity were calculated by PROBIT analysis based on percentage of mortality obtained at each concentration of the samples. EC_50_ is half maximal effective concentration. Complete statistical analysis was performed by means of the MicroCal Origin 6.0 statistics and graphs PC program.

## 4. Conclusions

Three new nordrimanes were synthetized from drimenol (1) and were compared with 11 sesquiterpenes and four nordrimanes. The new epoxide 12 presented an EC_50_ value of 83.2 ± 3.5 mg/L against *Drosophila melanogaster til-til*. In this work polygodial (2) did not show effective antifeedant activity against that biological target. The most active compound was isodrimenin (4) which presented a higher larvicidal activity of 4.5 ± 0.8 mg/L.
